# Effects of light-emitting diode spectral combinations on growth and quality of pea sprouts under long photoperiod

**DOI:** 10.3389/fpls.2022.978462

**Published:** 2022-09-07

**Authors:** Siqi Zhang, Xiaolei Guo, Junyan Li, Yinghua Zhang, Youming Yang, Wengang Zheng, Xuzhang Xue

**Affiliations:** ^1^National Research Center of Intelligent Equipment for Agriculture, Beijing, China; ^2^College of Agronomy and Biotechnology, China Agricultural University, Beijing, China

**Keywords:** pea sprout, LED spectral combination, growth, nutritional quality, plant factory

## Abstract

Pea sprouts have rich nutrition and are considered good for heart health. In this study, the kaspa peas and black-eyed peas were chosen to clarify the effect of different LED spectral combinations on the growth, yield, and nutritional quality of pea sprouts under long photoperiod (22 h light/2 h dark). The results showed that the two pea varieties responded differently to light spectral combinations. Black-eyed pea sprouts had higher plant height, fresh weight per plant, dry weight per plant, soluble sugar content, and lower malondialdehyde (MDA) content than kaspa peas under the same light treatment. Compared with white light, red-to-blue ratio of 2:1 significantly increased peroxidase (POD) and superoxide dismutase (SOD) activity, soluble sugar and soluble protein content of kaspa pea sprouts, and decreased MDA content of black-eyed pea sprouts. Blue light was negatively correlated with the plant height of pea sprouts and positively correlated with SOD activity, vitamin C, soluble sugar, and soluble protein content. Antioxidant capacity, yield, and nutritional quality of black-eyed pea sprouts were higher than those of kaspa pea sprouts under the same light treatment. Blue light improved the nutritional quality of pea sprouts. Compared with other light treatments, the red-to-blue ratio of 2:1 was more conducive to improving the antioxidant capacity and nutritional quality of pea sprouts under long photoperiod.

## Introduction

With the improvement of people’s living standards, healthy diet has attracted more and more attention. Vegetables are rich in nutrients needed by humans, and demands for vegetable yield and quality are getting higher (Kearney, 2010). Sprouts, which means the edible vegetables after seed germination (Benincasa et al., 2019), accumulate lots of bioactive substances, such as vitamins, polysaccharides, proteins and polyphenols (Gan et al., 2017). Sprouts are considered to have the function of anti-oxidation, anti-virus and anti-inflammatory, and reduce risk of diseases (Geng et al., 2022). As a high-quality sprout vegetable, pea sprout is rich in vitamin C and protein, and conducive to improve human immunity (Zhao et al., 2020). Sprouts also have the advantages of short growth cycle, low production cost, and small land area needed (Zhang et al., 2020).

A plant factory is a closed plant production system that aims to achieve high-precision control of plant growth environment and high crop yield (Avgoustaki and Xydis, 2020). Plant factory can avoid the impact of outdoor extreme weather conditions on plant growth (Al-Kodmany, 2018). Light-emitting diode (LED) is an indispensable lighting system that can provide different light quality, light intensity, and photoperiod to control plant growth. Compared with traditional lighting equipment, LED has the advantages of small volume, low energy consumption, long service life, safe use, energy, and environmental protection (Olle and Virsile, 2013).

Light is not only the energy for photosynthesis but also a signal for plant morphogenesis (Chen et al., 2017). Light quality and photoperiod have an important impact on plant growth and development, as well as yield and quality. Photoperiod can regulate plant germination, growth, and flowering (Cao et al., 2014; Bian et al., 2015). Previous studies have shown that long photoperiod can promote flowering of horticultural crops, shorten growth time of wheat, and increase leaf area of many temperate grass species (Adams and Langton, 2005; Sulpice et al., 2014; Watson et al., 2018). But excessive long illumination time will lead to reduction of yield, chlorosis of leaves, and weakened growth activity in tomatoes, cucumbers, and wheat (Gonzalez et al., 2003; Lanoue et al., 2021a). Some studies found that suitable light quality can reduce the adverse effects of long photoperiod on crops (Lanoue et al., 2021b).

Light quality affects plant height, chlorophyll content, antioxidant capacity, yield, and quality (de Wit et al., 2016; van Gelderen et al., 2018). Red and blue light is the main absorption wavelengths of plants (Ptushenko et al., 2020). Studies found that red LED can promote seed germination and plant elongation (Sellaro et al., 2012), and blue LED influences phytochrome content and stomata conductance (Samuoliene et al., 2013). Compared with monochromatic light, a mixture of red and blue LEDs can stimulate various types of photoreceptors in plants and reduce light stress (Sabzalian et al., 2014). However, optimal red-to-blue ratio varies among different plant species, even among different cultivars of the same plant species.

Black-eyed pea (Vigna unguiculata), also known as southern pea, cow pea, and crowder pea, grows well in summer and is native to Asia and Africa and contains significant amounts of protein, calories, and some water-soluble vitamins (Bhandari et al., 2016). Kaspa pea is a semi-leafless field pea variety that flourishes well in cool seasons (Sudheesh et al., 2015). This study was carried out in a plant factory to determine growth, yield, and nutritional quality of pea sprouts of the two cultivars under different LED spectral combinations and long photoperiod (22 h light:2 h dark).

## Materials and methods

### Experimental design

**FIGURE 1:**
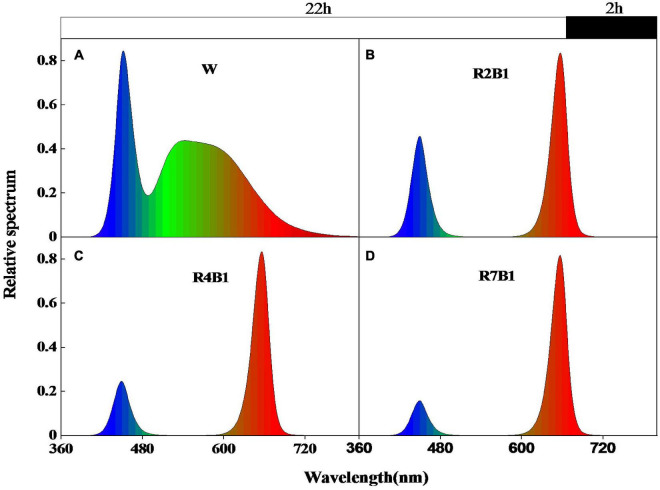
Relative spectrum distribution of the four LEDs’ light quality combinations. **(A)** White light (W); **(B)** red-blue light ratio 2:1 (R2B1); **(C)** red-blue light ratio 4:1 (R4B1); **(D)** red-blue light ratio 7:1 (R7B1). The white box above the figure represents the light duration of 22 h, and the black box represents the dark time of 2 h.

The experiment was conducted in the plant factory of the national precision agriculture experimental station in Xiaotangshan Town, Changping District, Beijing in 2019. Two pea cultivars, kaspa peas and black-eyed peas, were grown under four LED spectral combinations, including white light (W), red-to-blue ratio 2:1 (R2B1), red-to-blue ratio 4:1 (R4B1), and red-to-blue ratio 7:1 (R7B1). The relative spectral distribution (Figure 1) and relative spectral content (Table 1) were measured with a plant light analyzer (PLA-20, Everfine, Hangzhou, China), and the photosynthetic photon flux density (PPFD) was kept at 190 ± 25 μmol⋅m^–2⋅^s^–^^1^.

**TABLE 1:** Relative spectral content of the four LEDs’ light quality combinations.

Light quality treatment	Blue light (%)	Green light (%)	Red light (%)
W	34.06	43.61	22.32
R2B1	35.64	0.42	63.93
R4B1	22.14	0.47	77.38
R7B1	15.87	0.38	83.74

### Plant growth condition

Seeds were soaked in clean water for 12 h and then were sown in four sprouters (32 cm × 25 cm). One-half of each sprouter was occupied by 40 seeds of kaspa peas, and another half was occupied by 40 seeds of black-eyed peas. The four sprouters were covered with wet germination paper and put into a constant temperature incubator at 22^°^C for dark germination for 3 days. The four sprouters were then transferred under four LED light panels in the plant factory for hydroponic culture, with one sprouter under one LED spectral combination. The temperature in the plant factory was kept at 21 ± 1^°^C/17 ± 1^°^C (day/night), the photoperiod was 22 h light: 2 h dark, and the hydroponic nutrient solution was updated every week (Hoagland and Arnon, 1950). The distance between the bottom of the sprouters and the LED panels was 40 cm initially and adjusted during the experiment to maintain approximately stable PPFD (190 ± 25 μmol m^–2^ s^–1^) at the top of sprouts.

### Measurement of growth characteristics

After 12 days of LED light treatment, six plants from each cultivar in each sprouter were selected for measurement of growth characteristics. The remaining plants were stored in a –80°C ultra-low temperature freezer for measurement of antioxidant capacity and nutrition parameters. SPAD values of the upper three leaves of the six plants were determined by a chlorophyll meter (SPAD-502; Minolta, Osaka, Japan). Plant height of the six plants was measured using a ruler, and fresh weight was measured with one ten-thousandth scale (JA1003; balance instrument, Shanghai, China). After sterilizing the six plants at 105^°^C in a blast drying oven (DHG-9140a; Lakebo Instrument, Beijing, China) for 1 h, they were dried at 80^°^C to constant weight, and then the dry weight was measured by an electronic scale.

### Antioxidant capacity

The plants were taken out from the ultra-low temperature freezer and ground into powder in liquid nitrogen. Three samples of fresh tissue (0.2 g) from each treatment were homogenized with 50 mmol/L phosphoric buffer (pH 7.8), then centrifuged at 15,000 r/min for 15 min, and the supernatant (enzyme solution) was extracted for POD, CAT, and SOD activity determination.

POD and catalase (CAT) activities were determined by the guaiacol method (Liang et al., 2003). The POD activity was determined by the change rate of the absorbance value at 470 nm of the mixed assay of 0.05 ml enzyme solution, 2 ml 0.2% (w/v) H_2_O_2_, 0.95 ml 0.2% (w/v) guaiacol, and 1 ml pH7.0 phosphoric buffer; 1 ml 0.2% (w/v) H_2_O_2_ and 1.9 ml distilled water were added into 0.1 ml extracted solution, and the absorbance at 240 nm was measured to determine the CAT activity. The change of absorbance value of 0.01 per minute was token as an enzyme activity unit (U).

SOD activity was determined by nitroblue tetrazole (NBT) photochemical reduction method (Hassan et al., 2005). The 3 ml assay mixture contained 2.5 ml 13 μmol methionine, 0.25 ml 63 μmol/L NBT, 0.15 ml 13 μmol/L riboflavin, 0.05 ml phosphate buffer (pH 7.8), and 0.05 ml enzyme solution. Then it was exposed to light in a 4000-lx light incubator for 20 min, and the absorbance value at 560 nm was measured. One unit of enzyme activity was defined as inhibition of NBT photoreduction by 50%.

The content of malondialdehyde (MDA) was determined by thiobarbituric acid method (Al-Aghabary et al., 2004). Three samples of fresh tissue (0.3 g) from each treatment were homogenized in 0.5% (w/v) thiobarbituric acid and then heated in a boiling water bath for 10 min. After cooling, the homogenate was centrifuged at 3,000 *g* for 15 min. The absorbance value of supernatant was measured at 532, 600, and 450 nm, and the MDA content was calculated by the following formula:


MDA(mmol•gFW-1)=[6.452×(A-532A)600-0.559

×A]450×Vt/(V×sFW)

In the formula, V_*t*_ represents the total volume of the extract (ml), V_*s*_ represents the volume of the extract for measurement (ml), and FW represents the fresh weight of the sample (g).

### Nutrition parameters

The content of vitamin C was determined by molybdenum blue colorimetry (Li, 2000). Three samples of fresh tissue (0.2 g) from each treatment were homogenized with 5 ml oxalic acid-EDTA solution, then centrifuged at 3,000 *g* for 10 min. The assay mixture contained 1 ml supernatant, 5 ml oxalic acid EDTA, 0.5 ml metaphosphoric acid-acetic acid, 3.5% (w/v) H_2_SO_4_, and 5% ammonium molybdate, then the absorbance value was determined at 760 nm, and the content was calculated by a standard curve.

The content of soluble sugar was determined by the anthrone-sulfuric acid method (Fairbairn, 1953). Three dry samples (0.3 g) from each treatment were added to 5 ml distilled water and heated in an 85^°^C water bath and then centrifuged at 3,500 g for 10 min. After 5 ml anthrone and sulfuric acid were added to the 2 ml supernatant, the absorbance value at 620 nm was measured to determine the content of soluble sugar.

The content of soluble protein was determined by Coomassie brilliant blue G-250 method (Bradford, 1976). Three samples of fresh tissue (0.2 g) from each treatment were homogenized with 2 ml distilled water and centrifuged at 5,000 r/min for 10 min. Then 0.9 ml distilled water and 5 ml coomassie brilliant blue G-250 solution were added to 0.1 ml of the supernatant. The absorbance at 595 nm was measured to determine the content of soluble protein.

### Statistical analysis

In plant factories, spatial variability of hydroponic nutrient solution is neglectable and spatial variability of micro-climate is restricted by uniform ventilation from ventilation wall. So spatial replications of LED light treatments are not needed, especially when the LED panels are at the same height in the same level. We regarded this experiment as a completely randomized design. Six plants per treatment were used to measure the growth characteristics, and the determination of antioxidant capacity and nutritional parameters was repeated three times. Excel 2010 was used for data entry and processing. Variance analysis and principal component analysis were conducted using SPSS Statistics 25.0. The comprehensive scores in the principal component analysis of each treatment were calculated through the formula “comprehensive score = variance contribution rate of PC1 × variance contribution rate of FAC1 + PC2 × variance contribution rate of FAC2 + PC3 × variance contribution rate of FAC3” (Li et al., 2022). We used raw data and Origin 2020 for correlation analysis. Graphpad prism 8.0 was used for the creation of histograms. Duncan’s multiple range test was used for multiple comparisons in this experiment.

## Results

### Effects of light spectral combinations on growth characteristics of pea sprouts

**FIGURE 2:**
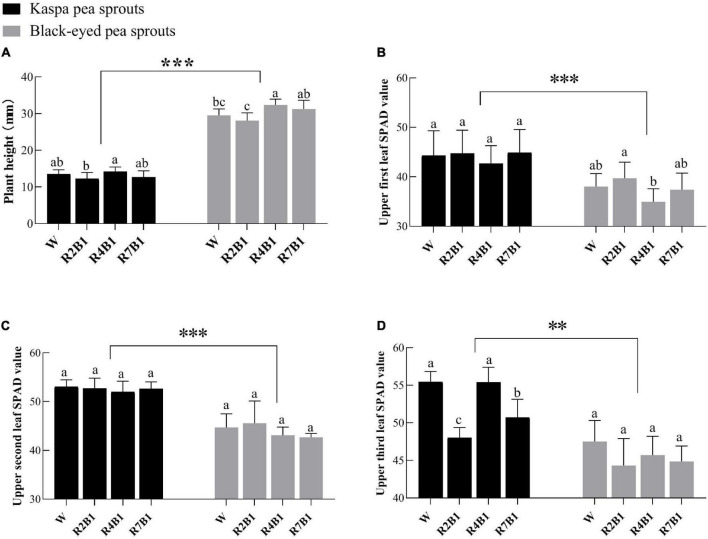
Effects of four LEDs’ light quality combinations on the plant height and SPAD value of the two cultivars of pea sprouts. **(A)** Plant height; **(B)** upper first leaf SPAD value; **(C)** upper second leaf SPAD value; **(D)** upper third leaf SPAD value. Different letters indicate significant differences at *P* ≤ 0.05 of the same variety among treatments. ** and *** denote significant differences at *P* ≤ 0.01 and *P* ≤ 0.001 of different varieties. Error bars represent six replicates.

As shown in Figure 2, the growth characteristics of the two pea sprout cultivars were significantly different. Plant height of black-eyed pea sprouts was significantly higher than that of kaspa peas, and SPAD values of the upper three leaves of kaspa peas were significantly higher than those of black-eyed peas. LED light treatment had significant effects on plant height and SPAD value of the upper third leaf and the interaction of variety and LED light treatment had a significant effect on the SPAD3 (Supplementary Table 1). Compared with white light, plant height of black-eyed pea sprouts increased significantly under R4B1. For the kaspa pea sprouts, plant height under R4B1 was slightly higher than that under white light, without significant difference (Figure 2A). There was no significant difference in SPAD values of the upper two leaves of kaspa pea sprouts among all the treatments, but the SPAD value of the third leaf under R2B1 and R7B1 was significantly lower than that under white light treatment. For black-eyed pea sprouts, the SPAD value of the upper three leaves showed little difference among different treatments (Figures 2B–D).

### Effects of different light quality combinations on antioxidant capacity of pea sprouts

**FIGURE 3:**
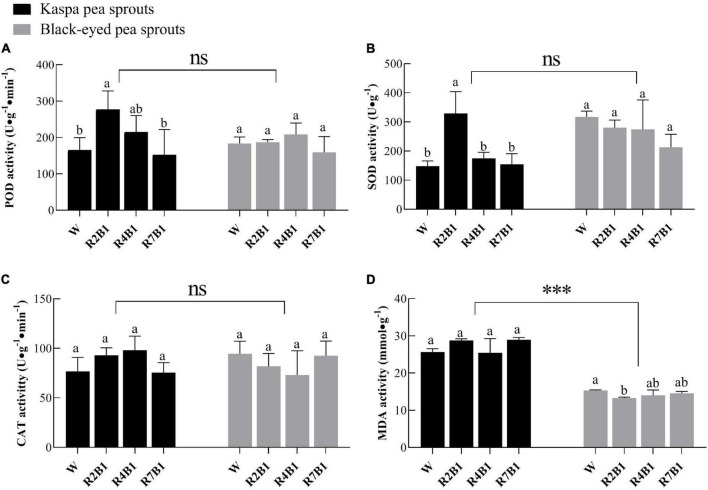
Effects of four LEDs’ light quality combinations on antioxidant capacity of two cultivars of pea sprouts. **(A)** Peroxidase (POD) activity; **(B)** superoxide dismutase (SOD) activity; **(C)** catalase (CAT) activity; **(D)** malondialdehyde (MDA) content. Different letters indicate significant differences at *P* ≤ 0.05 of the same variety among treatments. ***Denotes significant differences at *P* ≤ 0.001 of different varieties, and ns means no significant difference. Error bars represent six replicates.

Red and blue light ratios significantly affected the antioxidant capacity of pea sprouts (Figure 3). There was no significant difference in POD, SOD, and CAT activities between the two cultivars, while the MDA content of kaspa pea sprouts was two times higher than that of black-eyed pea sprouts. LED light treatment has no significant effect on CAT only and the interaction of variety and LED light treatment had a significant effect on antioxidant capacity (Supplementary Table 1). Activities of POD and SOD of kaspa pea sprouts were significantly higher under R2B1 than those under white light treatment, while there was no significant difference among different light treatments in activities of POD and SOD of black-eyed pea sprouts (Figures 3A,B). There was no significant difference in CAT activity between the two cultivars (Figure 3C). MDA content of black-eyed pea sprouts was significantly lower under R2B1 than that under white light. As the ratio of red light further increased, MDA content was still slightly lower than that under white light (Figure 3D).

### Effects of different light quality combinations on yield and quality of pea sprouts

**FIGURE 4:**
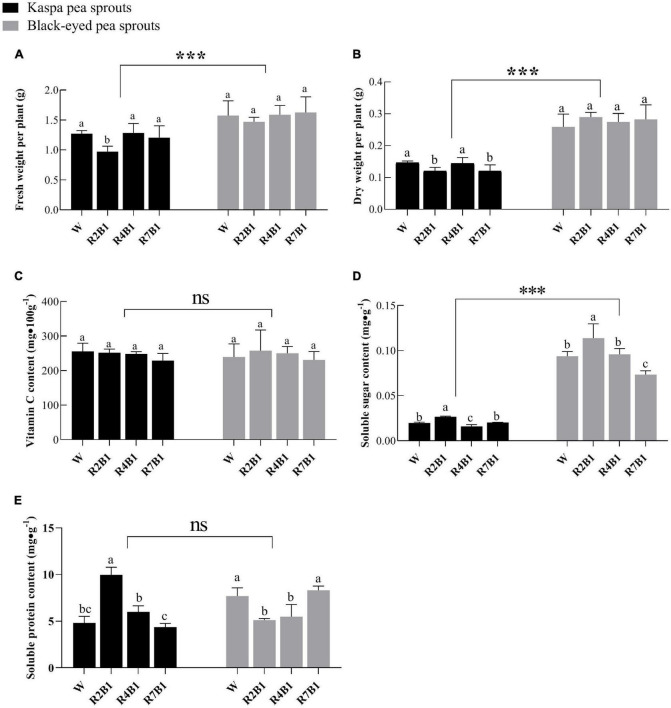
Effects of different light quality combinations on the yield and quality of two varieties of pea sprouts. **(A)** Fresh weight per plant; **(B)** dry weight per plant; **(C)** vitamin C content; **(D)** soluble sugar content; **(E)** soluble protein content. Different letters indicate significant differences at *P* ≤ 0.05 of the same variety among treatments. ***Denotes significant differences at *P* ≤ 0.001 of different varieties, and ns means no significant difference. Error bars represent six replicates.

As shown in Figure 4, fresh weight, dry weight, and soluble sugar content of black-eyed pea sprouts were significantly higher than those of kaspa pea sprouts, while there was no significant difference in vitamin C content and soluble protein content between the two cultivars. LED light treatment had significant effects on yield and nutritional quality except for dry weight, and the interaction of variety and LED light treatment had a significant effect on dry weight, soluble sugar content, and soluble protein content (Supplementary Table 1). Fresh weight of kaspa pea sprouts was significantly lower under R2B1 than that under white light, and dry weight of kaspa pea sprouts was significantly lower under R2B1 and R7B1 than that under white light. There was no significant difference in fresh weight and dry weight of black-eyed pea sprouts among different light treatments (Figures 4A,B).

There was no significant difference in vitamin C content between the two varieties. Soluble sugar content of the two varieties was significantly higher under R2B1 than that under white light. Soluble sugar content decreased with an increase in the proportion of red light, leading to the significantly lower soluble sugar content of kaspa pea sprouts under R4B1 than that under white light. Soluble protein content of kaspa pea sprouts was significantly higher under R2B1 than that under white light, but soluble protein content of black-eyed pea sprouts was significantly lower under R2B1 and R4B1 than that under white light.

### Principal component analysis of indicators of pea sprouts under different light quality combinations

Principal component analysis was carried out on 13 parameters including plant height, SPAD value of the upper three leaves, POD, SOD, and CAT activity, MDA content, fresh weight per plant, dry weight per plant, vitamin C, soluble sugar, and soluble protein content of pea sprouts under different light quality combinations. Three principal components PC1, PC2, and PC3 with eigenvalues greater than 1 were extracted, with contribution rates of 49.18, 17.35, and 9.27%, respectively, and a cumulative contribution rate of 75.80%. Plant height, SPAD value of the upper three leaves, MDA, fresh weight, dry weight, and soluble sugar had higher loadings on PC1, indicating that the PC1 basically reflected the information of these eight parameters. In addition, POD, SOD, and soluble protein had higher loadings on PC2, and CAT and VC have higher loadings on PC3. Therefore, these three principal components can be used to represent all indicators for analysis. The comprehensive score results showed (Table 2) that the score of R2B1 was the highest, which is 0.277, indicating that this treatment had the greatest impact on these three principal components. The second was R4B1, with a comprehensive score of 0.063, while R7B1 had the lowest score of –0.208.

**TABLE 2:** Factor analysis and comprehensive scores of 4 LEDs light treatments.

Treatments	FAC1	FAC2	FAC3	Composite scores
R2B1	0.024	0.780	0.667	0.277
R4B1	0.113	–0.003	0.200	0.063
W	–0.102	–0.189	–0.188	–0.132
R7B1	0.035	–0.639	–0.678	–0.208

FAC1, FAC2, and FAC3 represent the analysis factor scores of the three principal components, respectively.

### Correlation analysis between R: B ratio and various indicators of pea sprouts

**FIGURE 5:**
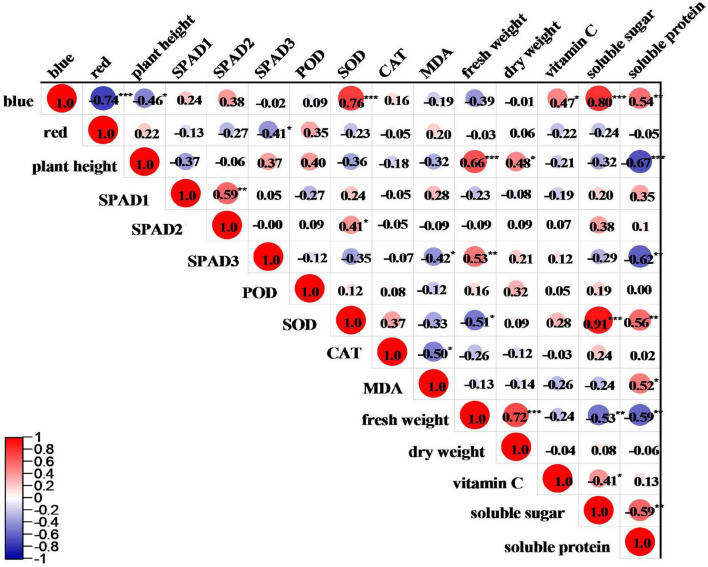
Correlation analysis between different light qualities and various indicators of pea sprouts. *, **, and ***Denote significant differences at *P* ≤ 0.05, *P* ≤ 0.01, and *P* ≤ 0.001 of different species. Numbers are correlation coefficients. SPAD1, SPAD2, and SPAD3 denote upper first leaf, upper second leaf, and upper third leaf. Blue and red, respectively, represent the percentage of blue and red light.

The correlations between R: B ratio and various indicators of pea sprouts are shown in Figure 5. Among the growth parameters, blue light was significantly negatively correlated with the plant height of pea sprouts, and red light was significantly negatively correlated with the SPAD value of the upper three leaves. Blue light was positively correlated with SOD activity. Red light was positively correlated with POD activity and MDA content but negatively correlated with SOD and CAT activity, and the correlations were not significant. There was no significant correlation between light quality and yield. Blue light was significantly and positively correlated with vitamin C, soluble sugar, and soluble protein. Red light was negatively correlated with vitamin C, soluble sugar and soluble protein, but the correlation was not significant.

## Discussion

With the emergence of LEDs, people can choose different wavelengths of light to regulate the growth of plants. Effects of light quality on vegetables such as lettuce, peppers, tomatoes, and cucumbers have been extensively studied (Li and Kubota, 2009; Su et al., 2014; Kaiser et al., 2019). Red and blue light have an important impact on plant height, chlorophyll, biomass, and photosynthetic capacity (Son and Oh, 2013; Liu and van Iersel, 2021; Liu et al., 2022). A mixture of red and blue light is more conducive to plant growth and development than monochromatic light, but the optimal ratio varies by plant species (Yu, 2021).

In this study, the plant height, antioxidant capacity, yield, and nutritional quality of black-eyed pea sprout were higher than those of kaspa pea sprout, indicating that the effect of light quality on plants was influenced by genotypes. Therefore, in production, it is not only necessary to select optimal light quality but also to choose suitable varieties according to the practical needs. The growth characteristics of pea sprouts under different ratios of red to blue showed that the plant height of pea sprouts was higher under R4B1 than that under white light (Figure 2). This was related to the fact that blue light was significantly negatively correlated with the plant height of pea sprouts in the correlation analysis (Figure 5). Therefore, the plant height of sprouts was higher when the ratio of red to blue was low, which was consistent with the previous study (Inoue and Kinoshita, 2017). However, the results of the principal component analysis showed that the comprehensive score of R7B1 was the lowest, indicating that it had the least impact on the indexes. Therefore, the plant height of sprouts was the highest under R4B1 instead of R7B1.

Chlorophyll is an important pigment for photosynthesis in green plants, so its content is significantly related to plant growth. The results showed that the SPAD value of the upper three leaves of pea sprouts decreased or did not change significantly under different ratios of red to blue light compared with white light (Figure 2). According to previous research reports, blue light can promote the synthesis of chlorophyll because blue light can induce the expression of key genes for chlorophyll synthesis (Liu et al., 2022). But red light reduces chlorophyll content because red light reduces 5-aminolevulinic acid, a precursor to chlorophyll synthesis (Sood et al., 2005). Therefore, the higher proportion of red light than blue light in the treatment of this experiment may be the reason why the SPAD value does not increase.

Under aerobic conditions, the production of reactive oxygen species (ROS) will hurt plant cells. In order to avoid ROS damage, plants have evolved defense systems to eliminate free radicals, including antioxidant and antioxidant enzyme systems (Logan et al., 2006). POD, SOD, and CAT are the main components in the plant antioxidant enzyme system and play important roles in regulating cellular redox state and responding to adverse environmental conditions (Loi et al., 2021). MDA is one of the most important products of membrane lipid peroxidation, and its content can indirectly reflect the degree of damage to the membrane system and the strength of plant stress resistance (Yamauchi et al., 2008). A number of studies have shown that blue light can improve the activity of antioxidant enzymes in many plants (Xu et al., 2014; Ye et al., 2017; Yu et al., 2017). Consistent with the above results, this study indicated that blue light was positively correlated with antioxidant enzyme activities (Figure 5), and R2B1 significantly enhanced POD and SOD activities of pea sprouts (Figure 4). Compared with white light, R2B1 reduced the MDA content of black-eyed pea sprouts and enhanced the antioxidant capacity of pea sprouts. Cryptochrome absorbing blue light can transmit the signal of stress response and then resist the damage of ROS by improving the activity of antioxidant enzymes (Jourdan et al., 2015).

The differences seen in growth, yield, and nutritional qualities between the two cultivars and among the different light treatments are not only a result of the light qualities tested. In this study, fresh weight and dry weight of kaspa pea sprouts were significantly lower under R2B1 than that under white light, which was consistent with a decrease of SPAD value in leaves under R2B1 (Figure 2). Results of previous studies on the effect of light quality on plant nutritional quality are contradictory. Some studies believe that the content of soluble sugar, soluble protein, and vitamin C in pea sprouts was higher when the R:B ratio was 3:1 or higher (Geng et al., 2017; Ban et al., 2019), while others found that the quality of tomato is better when blue light accounts for 60% of total light (Liu et al., 2010). In the study of tomato by Dong et al. (2019), it was found that the sugar content was the highest when the ratio of red to blue was 3:1, because light quality regulated the expression of protein genes related to glucose metabolism. In addition, studies have found that the soluble protein content of radish was higher under blue light than red light, because the nitrate reductase activity was stronger under blue light, which improves the nitrogen assimilation rate of plants (Maevskaya and Bukhov, 2005). However, it was also found that strawberry had higher contents of soluble protein, total sugar, and anthocyanin under red light (Peng et al., 2020). This study found that the content of soluble sugar and soluble protein of kaspa pea sprouts was significantly higher under R2B1 than that of white light, but the soluble protein content of black-eyed pea sprouts decreased under this treatment (Figure 4). Therefore, it can be seen that the effect of light quality on crop nutritional quality varies with different crop types and varieties.

## Conclusion

Black-eyed pea sprouts had higher plant height, while kaspa pea sprouts had higher SPAD values. Compared with white light, R4B1 significantly increased plant height of black-eyed pea sprouts and slightly increased plant height of kaspa pea sprouts.

MDA content of black-eyed pea sprouts was significantly lower than that of kaspa pea sprouts. R2B1 was optimal to improve POD and SOD activities of kaspa pea sprouts and reduced the MDA content of black-eyed pea sprouts. Antioxidant enzyme activities of black-eyed pea sprouts were not significantly affected by light treatments.

Black-eyed pea sprouts had a higher yield and soluble sugar content than kaspa pea sprouts. Compared with white light, R2B1 reduced the yield but increased the content of soluble sugar and soluble protein of kaspa pea sprouts. Yield of black-eyed pea sprouts was not significantly affected by light quality treatment. R2B1 increased the content of soluble sugar but decreased the content of soluble protein of black-eyed pea sprouts.

Black-eyed pea sprouts have a higher yield and better nutritional quality than kaspa pea sprouts. Among different R:B ratios, R2B1 was the best spectral composition for the nutritional quality of pea sprouts, significantly increased antioxidant enzyme activity, reduced content of MDA, and increased content of soluble sugar of pea sprouts.

## Data availability statement

The raw data supporting the conclusions of this article will be made available by the authors, without undue reservation.

## Author contributions

SZ and XG performed the experiment, analyzed the data, wrote the manuscript, and equally contributed to the research. JL participated in the experiment. XX and YZ designed and supervised the research. YY and WZ reviewed and edited the manuscript. All authors have read and agreed to the published version of the manuscript.
