# Supplementary material for: Effects of light-emitting diode spectral combinations on growth and quality of pea sprouts under long photoperiod
Source: Front Plant Sci. 2022 Sep 7;13:978462. doi: 10.3389/fpls.2022.978462 (PMC9490185; doi:10.3389/fpls.2022.978462)
Supplement: Supplementary file 1 [file Data_Sheet_1.PDF]

Supplementary Table

Supplementary Table1 Variance analysis of growth and quality parameters of two varieties of pea sprouts under four LEDs light treatments (P value).

Variation source	df	Plant height	SPAD1	SPAD2	SPAD3	POD	SOD	CAT	MDA	FW	DW	VC	SSC	SPC
Variety	1	0.000^{***}	0.000^{***}	0.000^{***}	0.004^{**}	0.181^{ns}	0.199^{ns}	0.918^{ns}	0.000^{***}	0.000^{***}	0.000^{***}	0.775^{ns}	0.000^{***}	0.511^{ns}
LED light	3	0.001^{***}	0.744^{ns}	0.687^{ns}	0.006^{**}	0.005^{**}	0.000^{***}	0.849^{ns}	0.000^{***}	0.009^{**}	0.874^{ns}	0.016^{*}	0.000^{***}	0.000^{***}
Variety × LED light	3	0.139^{ns}	0.202^{ns}	0.196^{ns}	0.000^{***}	0.000^{***}	0.000^{***}	0.000^{***}	0.000^{***}	0.418^{ns}	0.033^{*}	0.500^{ns}	0.000^{***}	0.000^{***}

*, ** and*** denote significant differences at P ≤ 0.05, P ≤ 0.01 and P ≤ 0.001 of different varieties, and ns means not significant (P >0.05). SPAD1、SPAD2 and SPAD3 denote upper first leaf, upper second leaf and upper third leaf. FW denote fresh weight and DW means dry weight. SSC denote soluble sugar content and SPC denote soluble protein content.
